# Anaesthetic Management of a Spontaneous Haemorrhagic Stroke Following Severe Preeclampsia: A Case Report

**DOI:** 10.7759/cureus.113765

**Published:** 2026-08-01

**Authors:** Teresa Ribeiro Boneco, Maria Massá Castro, Filipa Lança

**Affiliations:** 1 Anesthesiology and Perioperative Medicine, Unidade Local de Saúde Santa Maria, Lisbon, PRT; 2 Anesthesiology, Centro Hospitalar Universitário Lisboa Norte, Lisbon, PRT

**Keywords:** anaesthesia, caesarean section, haemorrhagic stroke, intracranial hypertension, neurocritical care, obstetric emergency, preeclampsia, pregnancy

## Abstract

Haemorrhagic stroke (HS) during pregnancy, especially in the context of preeclampsia, is a rare but critical emergency that poses significant health risks to both the mother and the foetus. Even so, rapid recognition and appropriate management are of utmost importance to minimise unfavourable outcomes. We present a case of the management of HS in a pregnant woman with severe preeclampsia, resulting in favourable outcomes for both mother and neonate. A 39-year-old female (gravida 8, para 4) at 32 weeks and six days of gestation was admitted with sudden-onset severe headache, vomiting, hypertension (200/100 mmHg), inability to speak, right-sided hemiplegia, and proteinuria. Given a depressed level of consciousness (Glasgow Coma Scale 9), she was intubated following rapid sequence induction. Neuroimaging showed a left cerebellar vermis and hemisphere haemorrhage with marked mass effect and transtentorial and tonsillar herniation. Emergency decompressive craniotomy with haematoma evacuation was performed under total intravenous anaesthesia (TIVA), with intracranial pressure and severe hypertension managed accordingly. Intraoperative deterioration of foetal cardiotocography prompted an emergency caesarean section in the same theatre, without patient transfer. The neonate was delivered with Apgar scores of 6, 8, and 9; transient respiratory depression required supplemental oxygen and a three-day neonatal unit admission, with no mechanical ventilation. The mother was extubated on day three and discharged two weeks after admission with residual ataxic gait and dysarthria. Throughout the case, continuous foetal monitoring was essential to guide the timing of delivery, underlining the importance of early recognition of foetal distress and immediate delivery without delay. This case emphasises the crucial role of the obstetric anaesthesiologist as the central coordinator in a complex multidisciplinary scenario involving simultaneous neurosurgical and obstetric emergencies.

## Introduction

Stroke during pregnancy is a rare event, with an estimated incidence of 10 to 30 per 100,000 deliveries [1]. Over 50% of pregnancy-related strokes are haemorrhagic and frequently associated with hypertensive disorders of pregnancy, vascular malformations, and coagulopathies [2]. In the obstetric population, preeclampsia, characterised by new-onset hypertension and proteinuria after 20 weeks of gestation, is a known risk factor for haemorrhagic stroke [1,2]. In spite of recent advances in obstetric and neurological care, the management of haemorrhagic stroke complicating preeclampsia remains a challenge, since maternal neurological stabilisation and foetal well-being must be weighed against each other.

In these scenarios, the obstetric anaesthesiologist plays a critical role, responsible not only for the airway, but also for intracranial pressure control and foetal surveillance, whilst coordinating the neurosurgical and obstetric teams. To our knowledge, reports describing emergency posterior fossa decompressive craniotomy followed immediately by caesarean section in the same operating theatre, under a single anaesthetic, in a patient with severe preeclampsia-associated cerebellar haemorrhage, are scarce. We report a case of spontaneous cerebellar haemorrhagic stroke following severe preeclampsia at 32 weeks and six days of gestation, requiring emergency decompressive craniotomy followed by emergency caesarean section within the same operative setting, with favourable outcomes for both mother and neonate.

## Case presentation

A 39-year-old female (gravida 8, para 4) at 32 weeks and six days of gestation was admitted to the obstetric emergency department with sudden-onset severe bilateral temporal headache, vomiting, and hypertension (blood pressure of 200/100 mmHg at admission). On physical examination, she presented with inability to speak, right-sided hemiplegia, a heart rate of 100-110 bpm in sinus rhythm, an irregular respiratory pattern, and proteinuria on a urine sample. Her Glasgow Coma Scale score was 9 (E2V2M5), with eye opening to pain, incomprehensible sounds, and localising motor response on the left side only due to right-sided hemiplegia. The pregnancy had been followed up regularly, but without prior diagnosis of hypertensive disease.

**Figure 1:**
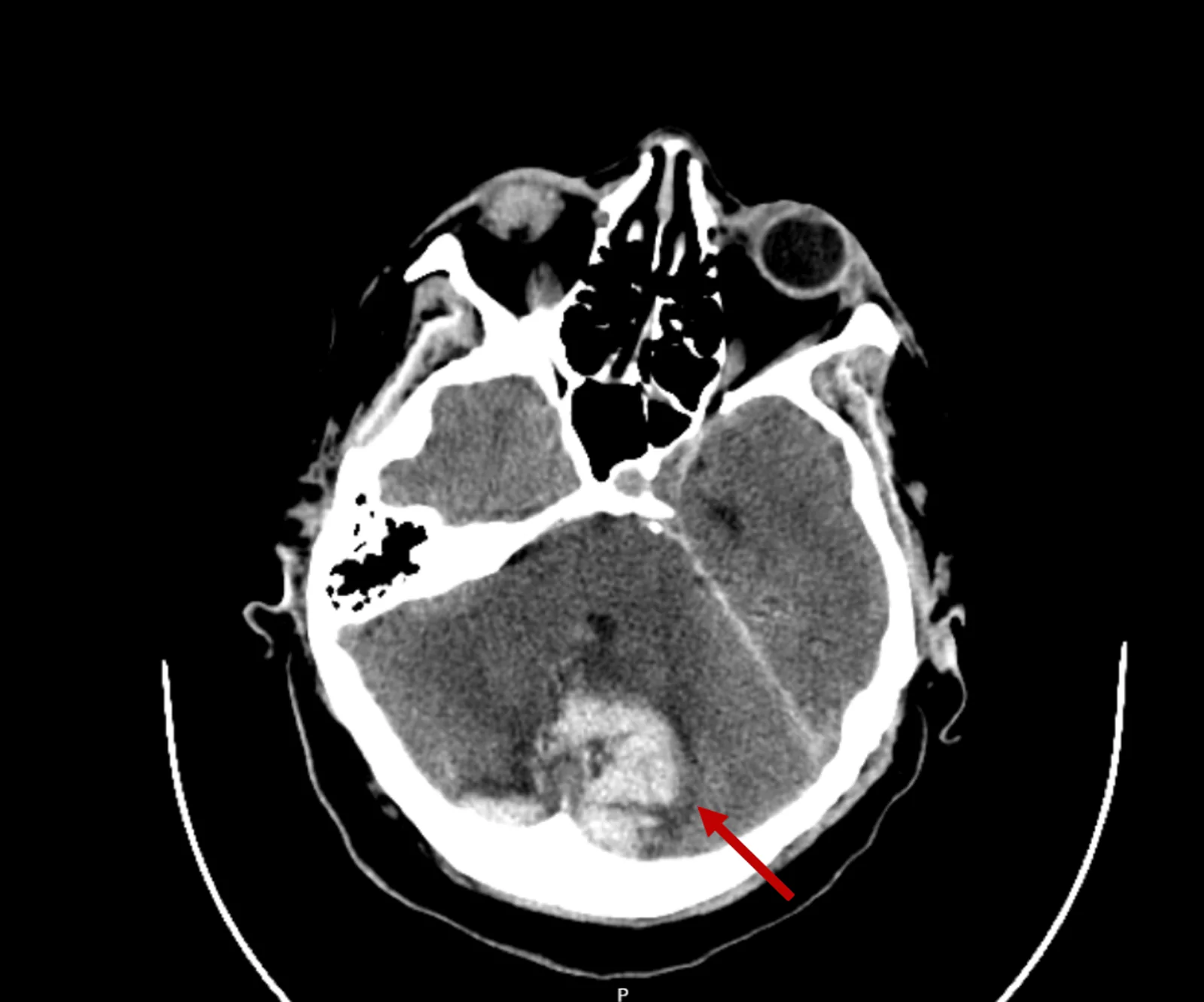
Axial CT scan of the brain Acute left cerebellar haemorrhage (arrow) with mass effect.

She was initially managed in a room within the delivery suite. To protect her airway, given her altered mental status, she was intubated following rapid sequence induction (RSI) with fentanyl 150 mcg, propofol 150 mg, and rocuronium 100 mg, and an arterial line was placed for continuous invasive blood pressure monitoring. Magnesium sulfate was started for eclampsia prophylaxis, with a 4 g loading dose followed by a 2 g/h infusion. Given the gestational age and the anticipated risk of preterm delivery, betamethasone was administered for foetal lung maturation during initial stabilisation. Initial foetal cardiotocography (CTG) was reassuring, with no evidence of foetal compromise at that time. Neuroimaging with computed tomography (CT) and magnetic resonance imaging (MRI) confirmed a left cerebellar vermis and hemisphere haemorrhage with marked mass effect and transtentorial and tonsillar herniation. Lead shielding was used to protect the foetus during CT imaging. The initial CT scan demonstrated the acute haemorrhage and the associated mass effect (Figure 1).

**Figure 2:**
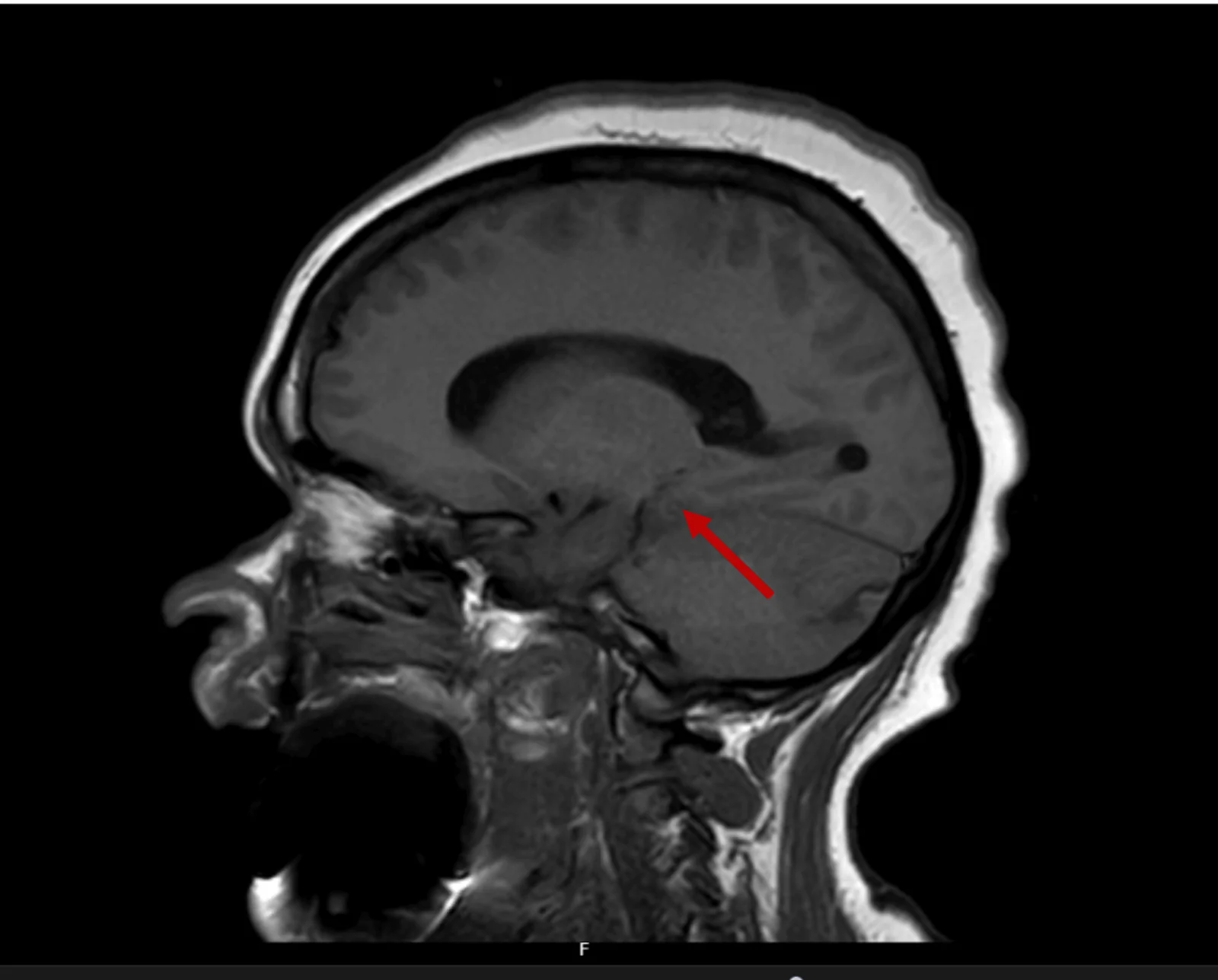
Sagittal T1-weighted MRI Posterior fossa showing transtentorial and tonsillar herniation (arrow).

**Figure 3:**
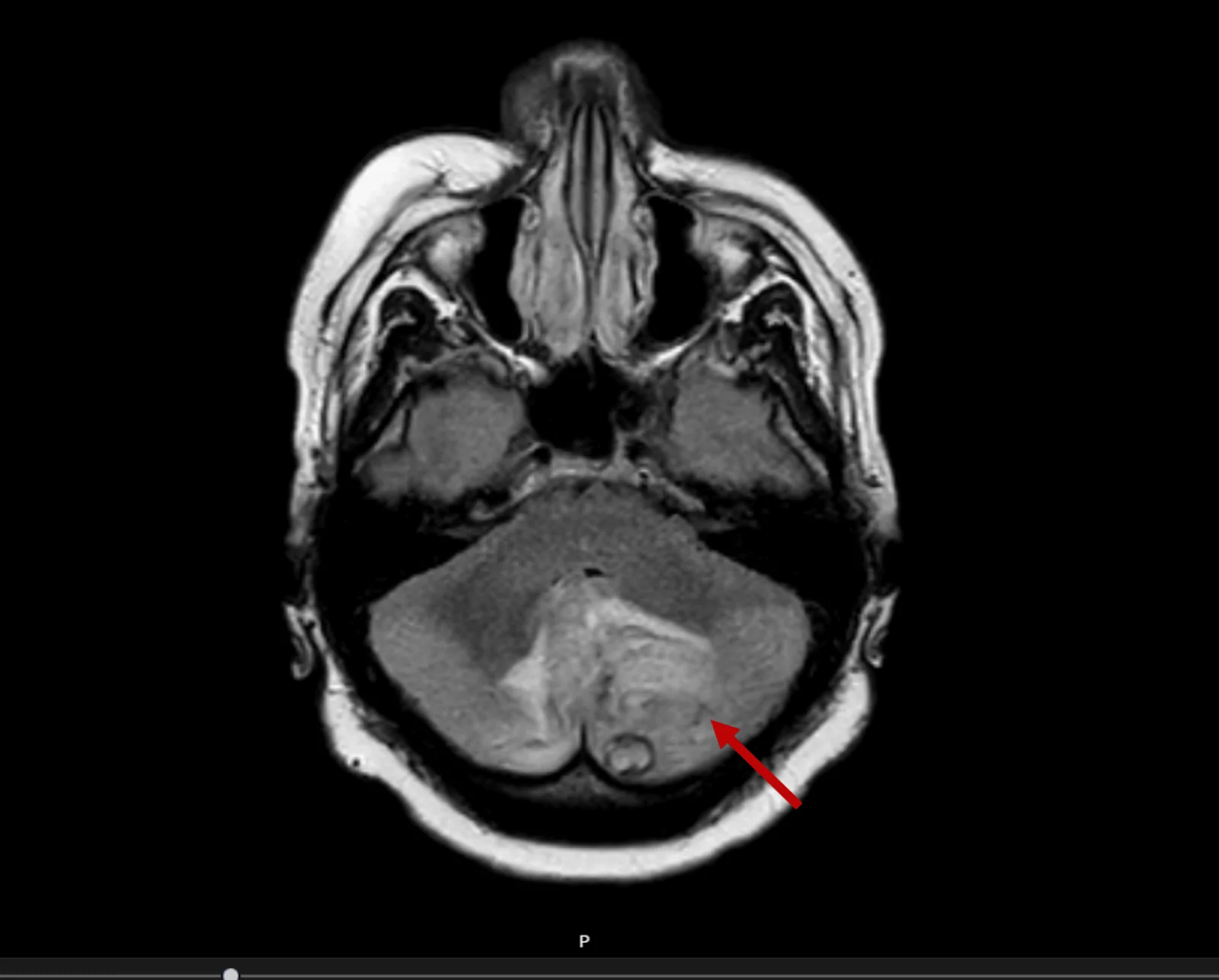
Axial MRI of the brain Left cerebellar haemorrhage (arrow).

MRI was performed in addition to CT to exclude an underlying structural or vascular cause, such as an arteriovenous malformation or cavernoma, which could have altered the surgical approach; this did not significantly delay the time to theatre. Sagittal MRI showed the posterior fossa with transtentorial and tonsillar herniation (Figure 2), and axial MRI further characterised the left cerebellar haemorrhage (Figure 3). Laboratory results and vascular imaging did not identify any alternative aetiology; the presumed cause was established as severe preeclampsia-associated hypertension by exclusion.

Approximately four hours after admission, she was taken to the theatre for emergency decompressive craniotomy with intraventricular catheter placement for continuous intracranial pressure monitoring and haematoma evacuation. As the patient was already sedated and intubated, and we anticipated she would need to remain sedated postoperatively, anaesthesia was maintained with total intravenous anaesthesia (TIVA) using propofol (4-8 mg/kg/h) and remifentanil (0.1-0.3 mcg/kg/min) infusions. Intracranial pressure was managed with mannitol (0.5 g/kg), hypertonic saline, and controlled hyperventilation guided by end-tidal carbon dioxide (EtCO2) and arterial blood gas partial pressure of carbon dioxide (PaCO2), targeting 30-35 mmHg (mild hypocapnia), to avoid uteroplacental vasoconstriction. A continuous intravenous labetalol infusion was used to control severe hypertension whilst maintaining adequate cerebral perfusion pressure.

During the craniotomy, foetal monitoring was continued, and an obstetrician present in the theatre identified deep and prolonged decelerations on the CTG tracing. An emergency caesarean section was therefore performed in the same operative setting under the existing general anaesthesia. The neonate was delivered with Apgar scores of 6, 8, and 9 at 1, 5, and 10 minutes. Transient respiratory depression was managed with supplemental oxygen; no mechanical ventilation was required. The infant was admitted to the neonatal unit for observation and discharged after three days.

Following surgery, she was transferred to the neurocritical care unit, where blood pressure was managed with a target systolic pressure of 140 mmHg and magnesium sulfate was continued for a further 48 hours postpartum. No antiepileptic prophylaxis was used, and a normocapnic ventilatory strategy was adopted, guided by continuous intracranial pressure monitoring. She was extubated on the third day of admission and discharged two weeks later with residual ataxic gait and dysarthria.

## Discussion

Over 50% of pregnancy-related strokes are haemorrhagic, frequently associated with hypertensive disorders, vascular malformations, and coagulopathies [2]. In this case, severe preeclampsia-associated hypertension was the presumed aetiology, as alternative causes were excluded by neuroimaging and laboratory results. Inadequate management of hypertension in pregnancy remains an important cause of preventable maternal morbidity and mortality [3].

In this case, airway management followed RSI principles: fentanyl was used to attenuate the hypertensive response to laryngoscopy, propofol for induction given its cerebral metabolic profile, and rocuronium provided neuromuscular blockade [4]. As the procedure was expected to be prolonged and we anticipated that the patient would need to remain sedated postoperatively, TIVA with propofol and remifentanil was the chosen option for anaesthesia maintenance. Neonatal respiratory depression was expected, given that remifentanil crosses the placenta; the neonate required supplemental oxygen and was kept under observation in the neonatal unit for three days. Intracranial pressure was managed with mannitol, hypertonic saline, and controlled hyperventilation targeting mild hypocapnia to avoid uteroplacental vasoconstriction. A continuous labetalol infusion was used to control severe hypertension whilst maintaining adequate cerebral perfusion pressure. Real-time foetal CTG monitoring throughout the neurosurgical procedure led us to perform an emergency caesarean section in the same operative setting, to protect both mother and foetus.

The timing of surgery is an important factor in cerebellar haemorrhage with mass effect and herniation. Posterior fossa haematomas have limited space to expand, and clinical deterioration from brainstem compression can be fast and hard to predict. Current guidelines recommend immediate surgical evacuation for patients with cerebellar haemorrhage who are deteriorating neurologically or who have brainstem compression or hydrocephalus, in order to reduce mortality [5]. Although evidence specific to cerebellar haemorrhage is limited, studies in supratentorial intracerebral haemorrhage with herniation suggest that earlier evacuation, within six hours of herniation onset, is associated with better survival and functional outcomes [6]. In this case, the patient presented with established herniation and a depressed level of consciousness, and surgical evacuation was carried out about four hours after admission, following airway management, invasive monitoring, and neuroimaging. The favourable maternal outcome, in spite of the severe presentation, supports the importance of timely and coordinated surgical management.

Improving prenatal education and interdisciplinary awareness of cardiovascular risks in pregnancy is essential. This case also shows how much the outcome depends on close communication between obstetricians, anaesthesiologists, and neurosurgeons.

## Conclusions

This case shows why pregnant women with neurological symptoms need a thorough neurological evaluation, and why obstetricians, anaesthesiologists, neurosurgeons, and neonatologists must work closely together to manage such complex cases. Stabilisation comes first, and when indicated, an emergency caesarean section should be performed without delay. Continuous foetal monitoring during any surgical procedure in a pregnant patient is essential, both to guide delivery decisions and to protect the mother and neonate.
